# Artificial Intelligence-Based Prediction of Molecular Alterations in Colorectal Cancer Using Routine H&E Whole-Slide Images

**DOI:** 10.3390/ijms27188025

**Published:** 2026-09-09

**Authors:** Marius Florentin Popa, Paul Șiancu, Călin-Ilie Mohor, Lilioara-Alexandra Oprinca-Muja, George-Călin Oprinca, Cosmin-Ioan Mohor, Ciprian Tănăsescu, Denisa Tănăsescu, Alina Cristian, Vicențiu-Vasile Vereș, Alina Bereanu

**Affiliations:** 1 Faculty of Medicine, Ovidius University of Constanța, 900527 Constanța, Romania; marius_popa2005@yahoo.com; 2 Faculty of Medicine, Lucian Blaga University of Sibiu, 550169 Sibiu, Romania; lilioaraalexandra.muja@ulbsibiu.ro (L.-A.O.-M.); georgecalin.oprinca@ulbsibiu.ro (G.-C.O.); cosmin.mohor@ulbsibiu.ro (C.-I.M.); tanasescuciprian@yahoo.fr (C.T.); denisa.tanasescu@ulbsibiu.ro (D.T.); alina.cristian@ulbsibiu.ro (A.C.); vicentiu.veres@ulbsibiu.ro (V.-V.V.); alina.bereanu@ulbsibiu.ro (A.B.)

**Keywords:** colorectal cancer, digital pathology, MSI phenotype, MMR status, BRAF, KRAS, artificial intelligence, hematoxylin and eosin

## Abstract

From a molecular perspective, colorectal cancer is a heterogeneous disease in which microsatellite instability (MSI) phenotypes, mismatch repair (MMR) status, chromosomal instability, or mutations in *BRAF*, *KRAS*, *NRAS*, and *TP53* can influence prognosis, hereditary cancer risk assessment, and therapeutic approaches. Conventional biomarker testing via immunohistochemistry, polymerase chain reaction, and next-generation sequencing remains the diagnostic standard, but it can be limited by cost, turnaround time, tissue consumption, and uneven access. This narrative review synthesizes 30 peer-reviewed studies, identified across major scientific databases, that evaluate artificial intelligence (AI) platforms designed to detect molecular alterations from routine hematoxylin and eosin-stained colorectal cancer tissue slides. The strongest and most reproducible evidence exists for MSI phenotypes and MMR status, for which weakly supervised, attention-based, transformer-based, foundation-model, and clinically oriented multiple-instance learning systems achieve high discriminatory performance and particularly high negative predictive values at screening thresholds. *BRAF* mutation status is moderately predictable, although often through morphology associated with microsatellite instability or the CpG island methylator phenotype (CIMP). In contrast, despite promising single-center results, the predictability of *KRAS*, *NRAS*, *PIK3CA*, and other point mutations remains less consistently generalizable. Interpretability analyses indicate that these algorithms rely on features such as tumor-infiltrating lymphocytes, plasma cells, mucinous and medullary differentiation, necrosis, stromal architecture, tumor heterogeneity, nuclear morphometry, and tumor purity. Current evidence supports the use of AI as a triage and enrichment tool rather than a replacement for validated molecular diagnostics. Ultimately, prospective validation, pre-analytical standardization, calibrated thresholds, regulatory oversight, and pathologist-centered workflow integration are essential for successful clinical translation.

## 1. Introduction

### 1.1. Molecular Pathways of Colorectal Cancer Development

**Figure 1:**
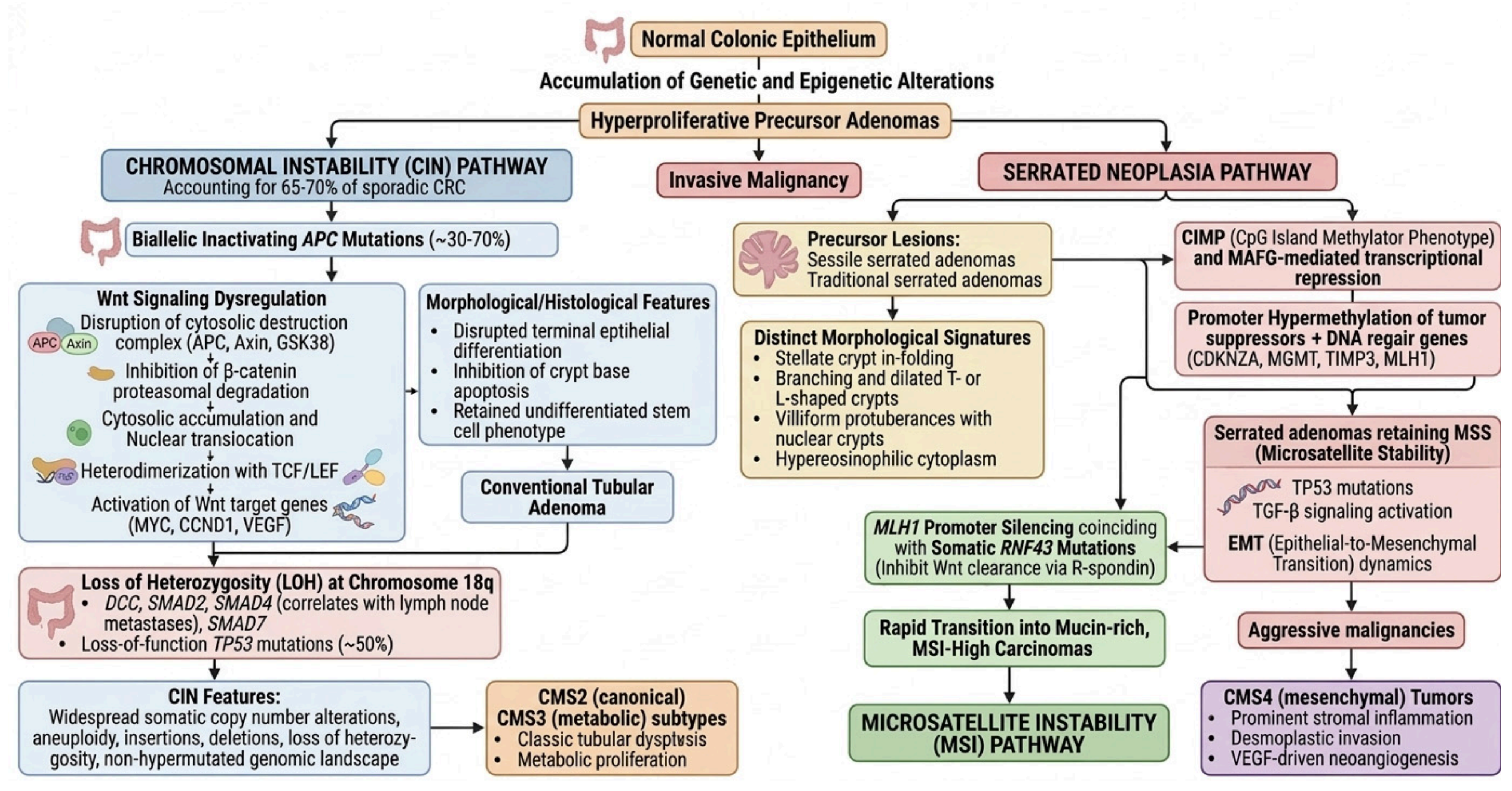
Molecular pathways and histological progression of colorectal carcinoma [1,2,3,4].

Colorectal carcinoma (CRC) pathogenesis proceeds through a multistep cascade driven by the sequential accumulation of genetic and epigenetic alterations. These alterations drive the histological progression from normal colonic epithelium to hyperproliferative precursor adenomas and, ultimately, invasive malignancy [1,2,3,4]. This genomic evolution segregates into three primary oncogenic trajectories: the chromosomal instability (CIN) pathway, the microsatellite instability (MSI) pathway, and the CpG island methylator phenotype (CIMP) or serrated neoplasia pathway [1,2,3,4] (Figure 1).

Accounting for 65% to 70% of sporadic CRCs, the CIN pathway is characterized by widespread somatic copy number alterations, aneuploidy, insertions, deletions, and loss of heterozygosity, generating a non-hypermutated genomic landscape [1,2,3,4]. The canonical initiating event occurs in 30% to 70% of sporadic tumors via biallelic inactivating mutations in the APC tumor suppressor gene. The disruption of the APC protein impairs the cytosolic multi-protein destruction complex—comprising APC, axin, and glycogen synthase kinase 3 beta (GSK3β)—thereby inhibiting the ubiquitin-mediated proteasomal degradation of β-catenin. The resulting cytosolic accumulation and nuclear translocation of β-catenin enable its heterodimerization with TCF/LEF transcription factors, constitutively activating Wnt target genes such as *MYC*, *CCND1*, and vascular endothelial growth factor (VEGF). Morphologically, this Wnt signaling dysregulation disrupts terminal epithelial differentiation and apoptosis at the crypt base, promoting an undifferentiated stem cell phenotype that initiates conventional tubular adenoma architecture [1,2,3,4] (Figure 1).

Alternatively, the serrated neoplasia pathway proceeds through precursor sessile serrated adenomas and traditional serrated adenomas. These display distinct morphological signatures, such as stellate crypt infolding, branched and dilated T- or L-shaped crypts, villiform protuberances with nuclear atypia, and hypereosinophilic cytoplasm [1,2,4]. This pathway frequently converges with CIMP, wherein MAFG-mediated transcriptional repression induces extensive promoter hypermethylation across tumor suppressor and DNA repair genes, including *CDKN2A*, *MGMT*, *TIMP3*, and *MLH1* [1,2,4].

### 1.2. Mismatch Repair Deficiency (dMMR) and the Microsatellite Instability (MSI) Phenotype

Hypermutated colorectal carcinoma (CRC) trajectories develop through defective DNA mismatch repair (MMR) machinery or DNA polymerase proofreading aberrations [2,5]. Deficient mismatch repair (dMMR) resulting in high microsatellite instability (MSI-H) accounts for approximately 10% to 20% of sporadic tumors and nearly all Lynch syndrome malignancies. Its prevalence is estimated at up to 20% in stage II and 12% to 15% in stage III disease before declining to roughly 4% to 5% in metastatic presentations. This phenotype arises from functional impairment of the DNA MMR machinery, driven either by germline pathogenic variants in the *MLH1*, *MSH2*, *MSH6*, or *PMS2* genes, or more frequently through sporadic epigenetic silencing via MLH1 promoter hypermethylation [1,2,3,4,5,6]. It is important to distinguish between these two deeply intertwined but distinct concepts: dMMR refers to the functional loss or structural deficit of mismatch repair proteins, whereas MSI refers to the resulting hypermutated genomic state. Currently, clinical “gold standards” for detection rely on these two distinct operational levels. The MMR state is routinely diagnosed via immunohistochemistry (IHC), which visualizes the loss of nuclear protein expression for MLH1, MSH2, MSH6, and PMS2. Conversely, the MSI phenotype is identified at the genomic level using multiplex polymerase chain reaction (PCR) to evaluate specific microsatellite markers (e.g., the Bethesda panel) or through next-generation sequencing (NGS) platforms that calculate tumor mutational burden (TMB) and microsatellite status [5,6].

Under physiological conditions, MLH1–PMS2 and MSH2–MSH6 heterodimers identify and correct DNA polymerase slippage errors within short, repetitive 1- to 6-nucleotide microsatellite motifs [5]. The failure of MutS and MutL enzyme complexes to excise insertion–deletion loops precipitates a hypermutated landscape. While specific diagnostic cutoffs vary by testing platform, this state is typically characterized by a high TMB (often exceeding 10–12 mutations/Mb) and a somatic mutation rate estimated to be 10- to 100-fold higher than that of MMR-proficient, microsatellite-stable (pMMR/MSS) neoplasms [1,2,5]. Before the advent of digital pathology and artificial intelligence (AI), gastrointestinal pathologists established a strong diagnostic basis for suspecting dMMR/MSI-H status directly from conventional hematoxylin and eosin (H&E)-stained whole-slide images. Morphologically, these hypermutated tumors frequently present with distinct histological hallmarks, most notably mucinous or medullary differentiation, a prominent inflammatory response characterized by dense tumor-infiltrating lymphocytes (TILs), and a pronounced Crohn-like peritumoral lymphoid reaction [5]. These classic, visually recognizable patterns provide the essential morphological foundation that allows deep learning algorithms to predict molecular alterations from routine H&E slides.

Ultimately, mismatch repair status is evaluated as a binary protein expression state (pMMR vs. dMMR), whereas microsatellite instability represents a continuum of genomic alterations, classically divided into high instability (MSI-H), low instability (MSI-L), and microsatellite stability (MSS). While dMMR strongly correlates with MSI-H and pMMR with MSS, they are not entirely synonymous. For instance, MSI-L tumors typically exhibit pMMR status but display minor genomic shifts that can confound purely morphological AI models. Furthermore, rare biological discordances (e.g., dMMR/MSS or pMMR/MSI-H) occur. Consequently, an AI algorithm trained to identify the histological footprints of genomic hypermutation (MSI-H) faces a fundamentally different classification task than one trained to predict the localized loss of protein expression (dMMR).

### 1.3. Driver Mutations in Colorectal Carcinoma

Subclonal progression across both chromosomal and microsatellite instability trajectories is propelled by specific driver oncogenes and complex signaling cross-talk, predominantly within the mitogen-activated protein kinase (MAPK) and phosphatidylinositol 3-kinase (PI3K)/AKT pathways. Activating missense mutations in *KRAS*, present in approximately 40% of sporadic cases, preferentially affect codons 12 and 13 of exon 2, codon 61 of exon 3, or codon 146. These alterations induce constitutive, epidermal growth factor receptor (EGFR)-independent downstream signaling through the RAF–MEK–ERK and NF-κB cascades [1,2,3,4]. In contrast, activating BRAF mutations, predominantly the V600E hotspot, are observed in approximately 10% to 15% of sporadic CRCs. While classically described as mutually exclusive with KRAS alterations, rare co-occurring mutations are occasionally documented in clinical practice, though they generally remain mutually exclusive at the dominant clonal level [3,4].

The MAPK signaling axis undergoes substantial cross-talk with the PI3K/AKT cascade, wherein activating missense mutations in *PIK3CA* occur in 25% to 32% of carcinomas to stimulate AKT/mTOR signaling. Together with *PTEN* inactivation and *TP53* disruption, these signaling aberrations drive uncontrolled proliferation, metabolic dysregulation involving glucose and amino acids, and apoptosis resistance via COX-2/PGE2 upregulation and FOXO1 inactivation. As previously described, deregulated TGF-β signaling and chromosome 18q loss of heterozygosity further dictate invasive behavior and metastatic progression [1,2,3,4].

Ultimately, the convergence of these driver mutations (*KRAS*, *BRAF*, *PIK3CA*), tumor suppressor alterations (*TP53*, *PTEN*, *APC*), and altered signaling cascades manifests as distinct, quantifiable histomorphological phenotypes on routine H&E whole-slide images. While CIN-driven tumors exhibit predictable, conventional tubular dysplasia and desmoplastic invasion, *BRAF*-mutant and MSI-H malignancies present with mucinous differentiation, medullary architecture, and dense peritumoral lymphocytic infiltrates [1,2,3,4,5,6].

## 2. Materials and Methods

A comprehensive literature search was conducted using the PubMed/MEDLINE, Web of Science and Embase databases to identify relevant studies published between 2021 and 2026, ensuring the inclusion of the most contemporary advancements in digital pathology. The search strategy was designed using a combination of free-text keywords and Medical Subject Headings (MeSH) structured around three core domains: artificial intelligence methodologies, histological modalities, and molecular biomarkers in colorectal cancer. The primary Boolean query employed was: (“artificial intelligence” OR “deep learning” OR “convolutional neural network”) AND (“digital pathology” OR “whole slide image” OR “H&E”) AND (“molecular” OR “MSI” OR “BRAF” OR “KRAS”) AND (“colorectal” OR “colon” OR “CRC”). The search results were sorted by best match and clinical relevance.

Initial screening of titles and abstracts was performed to establish study eligibility. Inclusion criteria mandated that studies: (1) utilized routine hematoxylin and eosin (H&E) stained whole-slide images; (2) applied deep learning or machine learning algorithms to predict specific molecular alterations or genomic phenotypes; and (3) focused strictly on colorectal carcinoma. Exclusion criteria were applied to remove non-English publications, reviews, non-human in vivo models, and purely computational computer science papers lacking direct histopathological interpretation. Following a rigorous full-text evaluation of the highly relevant subset, a final cohort of 30 peer-reviewed articles was purposefully selected to synthesize this narrative review.

## 3. Digital Pathology Frameworks for Molecular Prediction

Computational pathology has evolved rapidly from automated slide segmentation toward deep learning models capable of extracting complex prognostic and biological features from routine H&E slides. Broad systematic reviews of AI applications in colorectal cancer pathology have underscored this shift, highlighting how computer vision models automate tumor grading, tissue classification, and microenvironmental characterization to establish foundational digital workflows [7]. Building upon these image analysis frameworks, deep learning has increasingly been deployed to infer underlying genomic and epigenomic alterations directly from routine histopathology images. Recent systematic evaluations of mutation prediction models across solid cancers emphasize that while AI platforms can effectively capture morphological surrogates of driver alterations—including *KRAS*, *NRAS*, and *BRAF*—they face persistent translational hurdles, particularly regarding multi-center external validation, domain shift, and pre-analytical variability [8].

### 3.1. From H&E Whole-Slide Image to Molecular Probability

**Figure 2:**
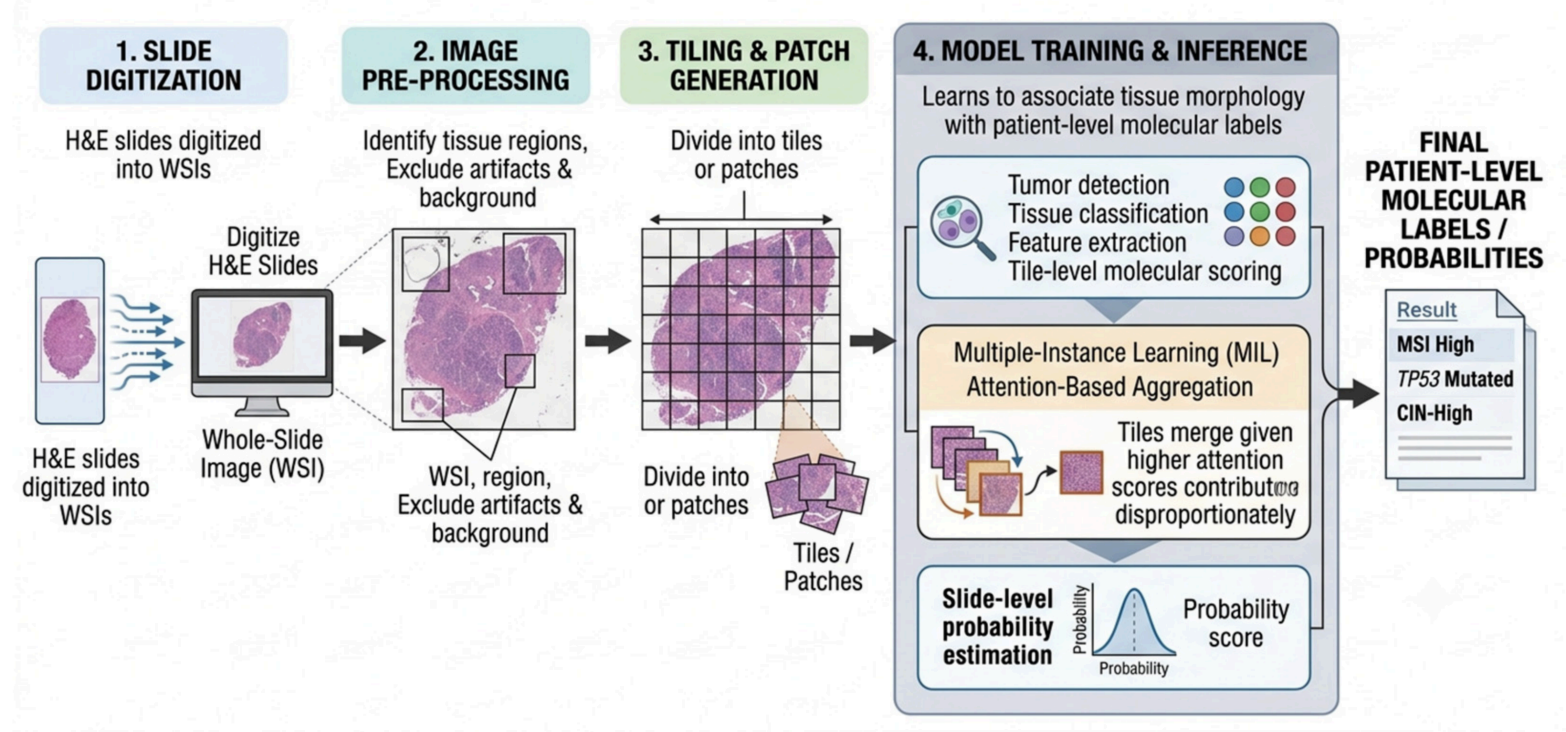
Digital pathology workflow for molecular prediction from H&E slides [14,15,16,17,18,19].

Most studies follow a convergent digital pathology workflow. H&E slides are digitized into whole-slide images (WSIs), diagnostically relevant tissue regions are identified, artifacts and background are excluded, images are divided into tiles or patches, and the model learns to associate tissue morphology with patient-level molecular labels. Depending on the study, the model may include tumor detection, tissue classification, feature extraction, tile-level molecular scoring, multiple-instance learning, attention-based aggregation, or slide-level probability estimation [9,10,11,12,13,14,15,16,17,18,19] (Figure 2).

Bilal et al. developed a weakly supervised framework using tumor detection, iterative draw-and-rank sampling, and HoVer-Net-based nuclear classification to predict hypermutation, MSI, CIN, CIMP-high, *BRAF*, *TP53*, and *KRAS* status from TCGA CRC slides [9]. Cao et al. proposed an Ensemble Patch Likelihood Aggregation model, linking MSI predictions to pathomic signatures and multi-omics interpretation [10]. Lou et al. introduced PPsNet, incorporating tumor detection and pair-wise learning to address intra-class variation and inter-class similarity in MSI prediction [11]. Chang et al. combined INSIGHT for tumor tile detection with WiseMSI, a self-attention-enabled convolutional network for slide-level MSI prediction [12]. Kim et al. summarized the PAIP 2020 challenge, which formalized MSI prediction and tumor area segmentation as benchmark tasks [13]. Later studies extended this framework through Swin Transformers, sequencer architectures, self-supervised learning, foundation models, and multi-scale multiple-instance learning [14,15,16,17,18,19] (Figure 2).

From a diagnostic pathology perspective, these pipelines reproduce a sequence of human reasoning: identifying diagnostically relevant tissue, distinguishing tumor from non-tumor structures, assessing tissue adequacy, evaluating biologically meaningful compartments and the tumor microenvironment, integrating heterogeneous regions, and, finally, issuing a probabilistic interpretation. The decisive difference is scale; AI can process thousands of fields across a WSI and quantify spatially distributed features that exceed the practical limits of human microscopic review.

### 3.2. Tumor Selection, Tissue Adequacy and Purity as Diagnostic Preconditions

Molecular inference from H&E is not independent of tissue adequacy. Factors such as tumor percentage, necrosis, mucin pools, inflammatory aggregates, tissue folds, cautery artifacts, poor focus, scanning differences, and biopsy fragmentation may alter model performance. Therefore, several studies incorporate tumor detectors or tissue classifiers into their workflows. Bilal et al. first separated tumor from non-tumor tiles before molecular prediction [9]. Chang et al. reported high tumor patch classification performance for INSIGHT and demonstrated a correlation between AI-derived and pathologist-estimated tumor cell fractions [12]. Guo et al. and Cen et al. used tissue classifiers trained on annotated CRC tissue categories to select tumor tiles prior to biomarker prediction [14,15]. Deepath-MSI introduced a minimum requirement of 100 tumor tiles, corresponding to approximately 6.6 mm^2^ of tumor area, to support stable predictions [17].

Schoenpflug et al. expanded this issue from AI model quality control to molecular pathology itself. Their SoftCTM approach quantified tumor purity at single-cell resolution in 1097 CRC patients, demonstrating high repeatability, strong correlations across paired H&E slides, and meaningful differences from conventional pathologist-derived tumor purity estimates and molecular deconvolution methods [20]. Most importantly, tumor purity estimation affected downstream copy number variation (CNV) calls, yielding discordant calls in approximately 6% to 13% of comparisons depending on the method [20]. For AI-based molecular prediction, this generates a core diagnostic principle: a model’s prediction is only as reliable as the tissue substrate and compartmental representation on which it is based.

### 3.3. Achieving Robust Generalization Through Large-Scale Datasets and Decentralized Training

The field has matured from single-cohort, proof-of-concept studies toward multicenter validation and data resource development. The SurGen dataset contributes 1020 H&E WSIs from 843 CRC cases with *KRAS*, *NRAS*, *BRAF*, and MMR status, as well as survival and staging annotations, supporting future research on biomarker development and prognostic modeling [21]. Similarly, the PAIP 2020 challenge provided MSI labels and tumor segmentation annotations, enabling algorithmic comparisons in a structured challenge format [13]. Furthermore, Saldanha et al. addressed the major obstacle of multicenter data sharing through swarm learning, training MSI and *BRAF* predictors across physically separated cohorts from Northern Ireland, Germany, and The Cancer Genome Atlas (TCGA), with external validation in UK cohorts [22]. Their results demonstrated that swarm learning could perform comparably to centrally merged models and outperform many local models, all while avoiding the direct transfer of sensitive patient data [22].

This represents more than a mere technical achievement. For successful clinical translation, AI models must generalize across different laboratories, slide scanners, patient populations, fixation protocols, staining platforms, and case mixes. Federated or decentralized machine learning approaches may therefore become essential for building robust pathology AI systems without compromising institutional data governance.

## 4. AI-Driven Prediction of MSI Phenotype and MMR Status

To contextualize the molecular targets evaluated by AI platforms, this study systematically addresses the full spectrum of MMR status alongside MSI phenotypes. Biologically, MMR status represents the overarching functional category of the DNA repair system—encompassing both pMMR and dMMR states at the protein level—whereas MSI denotes the downstream genomic expression pattern (MSI-High vs. MSS). Because dMMR constitutes a specific loss-of-function subset within the broader MMR pathway rather than a standalone diagnostic category, our framework evaluates AI prediction across both protein-level expression states and genomic instability patterns.

### 4.1. MMR Status and MSI Phenotypes as the Most Mature H&E-Based Molecular Prediction Sets

Among all molecular targets in CRC, MSI/dMMR has the strongest and most biologically plausible histopathological evidence. Its morphological correlates—such as tumor-infiltrating lymphocytes, a Crohn-like lymphoid reaction, mucinous differentiation, medullary morphology, poor differentiation, stromal plasma cells, and a right-sided phenotype—are well known to gastrointestinal pathologists, although they are insufficiently specific as stand-alone diagnostic criteria without molecular confirmation [9,17,19,23,24,25,26]. However, when evaluating AI platforms, it is critical not to conflate dMMR and MSI as a single computational target. Given that their clinical detection methods are fundamentally different, AI models trained on these distinct ground truths are predicting different biological phenomena. Models trained against an IHC reference standard are technically predicting the spatial absence of mismatch repair proteins (dMMR). In contrast, models trained against PCR or NGS reference standards are recognizing the downstream morphological consequences of genomic hypermutation (MSI). While these two conditions are highly concordant in clinical practice, grouping them together in algorithmic evaluations obscures important nuances, such as those cases exhibiting biological discordance (e.g., dMMR tumors that remain microsatellite stable). Therefore, an algorithm’s performance and its visual interpretability must always be contextualized by whether the machine learning architecture was optimized to detect protein loss or genomic instability.

The computational prediction of MSI and dMMR from routine H&E WSIs has progressed from early convolutional baselines to complex, multi-scale generative architectures. Initial proof-of-concept pipelines relied on dense manual tumor annotations and two-stage multiple-instance learning (MIL) frameworks, such as Ensembled Patch Likelihood Aggregation (EPLA), which utilized ResNet-18 feature extraction combined with patch likelihood histograms and bag-of-words classifiers to predict slide-level MSI status [10]. To overcome the bottleneck of dense, pixel-level labeling, subsequent methodologies transitioned toward weakly supervised learning paradigms. Frameworks such as Iterative Draw and Rank Sampling (IDaRS) coupled automated convolutional tumor detection with iterative scoring networks to project slide-level probabilities without fine-grained spatial annotations [9], while Parameter Partial Sharing Networks (PPsNet) implemented deep supervision with twin convolutional networks sharing shallow weights to minimize hardware memory loads and capture synergistic tissue variances [11]. As feature extraction backbones evolved beyond standard convolutional neural networks (CNNs), researchers explored efficient sequence architectures. One such structure is the digital pathology sequencer (DPSeq), which deploys two-dimensional bidirectional long short-term memory (BILSTM2D) blocks across color-normalized tiles, capturing long-range architectural context while bypassing the quadratic computational penalties of self-attention matrices found in standard vision transformers (ViTs) [15].

Recent architectural designs have increasingly converged on self-supervised foundation models and attention-based MIL (attMIL) to extract scale-invariant feature embeddings directly from uncurated slides. Contrastive learning encoders trained across tens of millions of pathology images—including RetCCL, CTransPath, DINOv2, and multimodal vision–language models such as CONCH, UNI, and HOptimus0—have largely supplanted ImageNet-pretrained backbones. These enable robust slide-level aggregation via clustering and prototypical networks without traditional stain normalization or manual tumor masking [16,18,19,27]. Expanding beyond macro-level patch classification, computational frameworks have incorporated subcellular and nuclear segmentation networks, deploying tools such as HoVer-Net, QuPath, and StarDist to isolate individual neoplastic nuclei [9,24,28]. These cytological pipelines quantify nuclear size variance through metrics such as the standard deviation of nuclear area [28], or apply single-cell genomics clustering algorithms to compute continuous tumor heterogeneity scores (THS) at the 95th percentile of pairwise tile cosine distances [24]. By fusing these sophisticated image matrices with clinical variables or multi-omic profiles—utilizing Multimodal Compact Bilinear (MCB) pooling or survival transformer platforms (MOMA) across DNA methylation, mRNA, and copy number variations—computational oncology has established that visual histopathological architectures act as direct spatial proxies for underlying CpG island methylation, MLH1 promoter silencing, and interferon cytolytic signaling cascades, which are key molecular pathways in MSI colorectal tumors [10,29,30,31].

### 4.2. Methodological Heterogeneity, Risk of Bias, and Metric Comparability in MSI and MMR Models

Direct cross-study comparison of reported predictive performance metrics (such as AUROC or F1-scores) is inherently limited by substantial methodological heterogeneity, spectrum bias, and variable reference standards across the literature. Studies vary significantly in how MSI/dMMR status is defined. Polymerase chain reaction (PCR) measures genomic microsatellite insertions, deletions, or known mutations in the MMR genes; next-generation sequencing (NGS) calculates continuous MSI sensor scores or tumor mutational burden; and immunohistochemistry (IHC) evaluates the nuclear protein expression of MLH1, MSH2, MSH6, and PMS2. Because biological discordance between IHC protein loss and molecular instability can occur, models trained on one modality may exhibit lower agreement when tested against another, reflecting underlying assay discordance rather than AI failure.

Benchmark performance is heavily conditioned on underlying training and testing populations. Public repositories such as TCGA predominantly contain flash-frozen or formalin-fixed paraffin-embedded (FFPE) resection specimens from Western populations, whereas Asian multicenter cohorts [11,12] exhibit different baseline tumor characteristics and pre-analytical tissue handling protocols. Furthermore, challenge datasets (e.g., PAIP 2020) [13] often pre-select high-quality, high-purity tumor regions, artificially inflating model accuracy compared to uncurated routine pathology workflows containing tissue folds, batch effects, or low tumor purity [20,23]. Receiver operating characteristic (ROC) curves and AUROC values are mathematically insensitive to class imbalance, making them poor indicators of clinical utility when deployed in low-prevalence screening settings. In stage II/III CRC, dMMR prevalence ranges from 12% to 20%, but it drops to 4% to 5% in metastatic disease [5,6]. Consequently, an AUROC exceeding 0.95 may yield a high positive predictive value (PPV) in a balanced challenge dataset, but a much more modest PPV (e.g., 34.7%) in a real-world, population-level screening pipeline where disease prevalence is low [17]. Performance metrics fall into distinct tiers of generalizability: internal cross-validation (highest risk of overfitting), local cohort fine-tuning, and true zero-shot external multicenter validation. Models achieving high internal AUROCs frequently suffer performance decay when deployed externally without domain adaptation or cross-domain testing [10,12].

Furthermore, the physical processing of tissue within the training datasets introduces a critical pre-analytical bias. As noted in the EPLA pipeline developed by Cao et al., early foundational models frequently relied on flash-frozen tissue sections provided by the TCGA database for training, primarily because these slides were readily paired with multi-omics data. However, flash-frozen tissue is not the clinical standard for morphological assessment or MSI determination [10]. Frozen sections exhibit significant morphological distortions, including nuclear ice crystal artifacts, cellular swelling, and altered stain affinities, which drastically diverge from the appearance of standard FFPE tissue. Consequently, an AI model trained to recognize MSI on frozen tissue cannot be directly deployed in a routine clinical laboratory without significant domain adaptation or transfer learning to account for the FFPE morphological shift. Future benchmark evaluations must prioritize models trained natively on FFPE datasets.

When evaluating the efficacy of these AI pipelines, it is imperative to clearly distinguish between the levels of validation, as performance estimates from different study designs are not directly comparable. Internal validation frequently overestimates model performance due to overlapping pre-analytical variables. External retrospective validation tests the algorithm on unseen, archived cohorts from different institutions, assessing generalized performance against distinct tissue preparation protocols and scanners. However, algorithms can be genuinely considered for clinical use only following prospective clinical validation, where the AI is deployed continuously within a live diagnostic workflow, facing unselected, consecutive cases. Because the transition from curated retrospective datasets to prospective clinical realities often introduces unexpected performance drops, AUROC or accuracy scores reported across these fundamentally different cohorts must be evaluated in strict isolation, rather than compared side-by-side.

### 4.3. Clinical Implementation of MSI Phenotype and MMR Screening Through AI Platforms

Translating computational pathology from in silico discovery to routine clinical workflows provides an actionable framework for pre-screening triage, universal Lynch syndrome surveillance, and neoadjuvant immunotherapy stratification.

Regarding the sensitivity and specificity of MSI/dMMR pipelines, early convolutional models demonstrated high internal accuracy. For example, EPLA achieved an area under the receiver operating characteristic curve (AUROC) of 0.88 on flash-frozen TCGA cohorts, yet it experienced performance attenuation when deployed on external formalin-fixed paraffin-embedded (FFPE) tissues, dropping to an AUROC of 0.85 before local fine-tuning [10] (Table 1). In contrast, weakly supervised architectures and efficient sequence backbones have demonstrated superior cross-cohort resilience. IDaRS achieved an external AUROC of 0.98 on unseen challenge datasets [9], while DPSeq reached AUROCs of 0.92 (area under the precision–recall curve [AUPRC] = 0.68) for MSI and 0.88 for hypermutation across international validation sets [15] (Table 1). Multi-target transformer encoders evaluated across 1912 patients similarly secured external AUROCs of 0.93 for MSI, 0.88 for hypermutation, and 0.78 for BRAF mutations. Hierarchical clustering demonstrated that predictive accuracy peaks for mutations co-occurring within specific genetic clusters sharing dominant morphological surrogates [27] (Table 1). Bustos et al. developed xDEEP-MSI, an explainable, bias-rejecting system trained on tissue microarrays (TMAs) from 1788 patients, reaching a patient-level AUROC of 0.90 while explicitly addressing batch effects related to project origin, patient spot, and TMA glass [23] (Table 1). Furthermore, Lou et al. reported a validation accuracy of 87.3% and an AUROC of 0.94 using PPsNet in an Asian CRC dataset [11] (Table 1). Chang et al. reported WiseMSI specificity of 94.7%, sensitivity of 84.7%, and an AUROC of 0.95 in a multicenter Chinese cohort, although cross-cohort testing on TCGA showed that performance remained sensitive to domain differences [12] (Table 1).

The PAIP 2020 challenge further established MSI prediction as a benchmark problem. The top-ranked system achieved an F1-score of 0.9231 using approaches that commonly combined CNN-based classification with tumor segmentation or tumor-aware preprocessing [13]. Transformer and efficient sequence-based architectures subsequently improved data efficiency. Guo et al. reported an AUROC of 0.91 for MSI using Swin-T in TCGA cross-validation, and an AUROC of 0.90 in cross-study validation using MCO for training and TCGA for testing [14]. Cen et al. reported strong DPSeq performance for MSI and other biomarkers, outperforming several CNN and transformer comparators under matched experimental conditions while requiring less training and prediction time [15] (Table 1).

The most clinically relevant metric for MSI/dMMR AI is not simply the AUROC. A diagnostic prescreening tool must operate at a clinically specified sensitivity threshold and minimize false-negative MSI/dMMR cases. For MSI, a high negative predictive value (NPV) is particularly important, as false-negative results could delay immunotherapy eligibility, hereditary cancer work-ups, or appropriate confirmatory testing. Niehues et al. evaluated multiple architectures in a realistic multicenter external validation and found that self-supervised, attention-based multiple-instance learning generalized well for MSI and BRAF, while KRAS, NRAS, and PIK3CA predictions remained below clinical-grade performance [16]. At a predefined high-sensitivity threshold, the MSI model achieved a high NPV, supporting its use as a rule-out prescreen rather than a definitive molecular replacement [16]. Feng et al. provide some of the most robust evidence in the current literature. Deepath-MSI was trained and validated on 5070 WSIs from seven cohorts, achieving an AUROC of 0.98 in the multicenter test set. At a fixed 95% sensitivity threshold, specificity was 91.7%, PPV was 69.0%, NPV was 99.0%, and accuracy was 92.2%. In a real-world validation cohort, the model maintained a sensitivity of 94.6%, a specificity of 90.7%, a PPV of 34.7%, and an NPV of 99.7% [17] (Table 1). This performance profile is clinically meaningful: AI-positive cases still require confirmatory IHC, PCR, or NGS, but AI-negative cases may be safely deprioritized in carefully validated workflows.

### 4.4. Foundation Models, Multi-Scale Models, and Non-Tumor Regions

Foundation models offer a new strategy by utilizing large, pretrained pathology encoders and adapting them to MSI prediction with minimal task-specific engineering. Bilal et al. benchmarked UNI, Virchow2, and CONCH for MSI prediction across the TCGA and PAIP cohorts. CONCH, coupled with two-cluster aggregation and ProtoNet adaptation, achieved the best balanced accuracy in external PAIP validation, whereas UNI performed best in TCGA cross-validation. Interestingly, a fine-tuned ResNet34 baseline outperformed the foundation models in a smaller internal PAIP validation setting [18]. This mixed result highlights an important caveat: while foundation models are promising, they are not automatically superior. The choice of adapter strategy, aggregation method, model calibration, and rigorous external validation remain decisive factors.

Petäinen et al. evaluated 24 modeling approaches, varying tissue origin, magnification, and embedding strategy. While tumor regions remained the most predictive, non-neoplastic tissue still contained a measurable dMMR signal. In external validation, the top foundation-model-based approaches achieved high F1-scores and specificity, although sensitivity varied substantially across cohorts [19]. This observation supports a broader histopathological concept: MSI/dMMR is not purely an epithelial tumor-cell pattern, but rather a complex tumor–host phenotype involving the immune response, stromal organization, and adjacent tissue context.

### 4.5. Multimodal Data Fusion, Hybrid Modeling, and Morphologic Heterogeneity in MSI Prediction

Several studies extend MSI prediction beyond H&E alone. Qiu et al. integrated H&E image features with mRNA, miRNA, lncRNA, DNA methylation, and CNV data using multimodal compact bilinear pooling. H&E alone achieved an AUC of 0.809 in fivefold cross-validation, while H&E combined with DNA methylation achieved the highest AUC of 0.952. However, combining H&E with all omics modalities decreased performance relative to selected single-modality fusions. This suggests that multimodal integration must be biologically and statistically disciplined rather than indiscriminate [29] (Table 2).

Wei et al. developed a hybrid MSI model integrating pathomic signatures with clinical features. A fully supervised pathology model outperformed a semi-supervised pathology model internally, while the hybrid model achieved an AUROC of 0.95 in validation and 0.86 in an external Dongyang cohort [31]. The model’s associated transcriptomic analysis identified MSI-related enrichment in immune pathways, including the humoral immune response, and in hub genes such as IFNG and CD8A [31]. These findings reinforce a recurrent observation across the corpus: MSI prediction is strongly tied to the structure of the immune microenvironment.

Rubinstein et al. approached MSI from the perspective of tumor heterogeneity. Their tumor heterogeneity score (THS), derived from deep learning imaging features, was significantly higher in MSI-H tumors than in MSS/MSI-L tumors, higher in MLH1-silent tumors than in non-silent tumors, and correlated with the MSIsensor score [24]. Slide-level direct MSI classification reached an AUROC of 0.7 ± 0.1, which is lower than that of dedicated MSI classifiers; however, the study is conceptually important because it spatially links MSI biology to intratumoral morphological diversity [24].

**Table 2:** Predictive Performance and Biological Impact of Targeted Multimodal Data Fusion in MSI and MMR Prediction.

Modality Combination	Model Performance (AUROC)	Clinical Implication
H&E Alone	0.80	Serves as a strong baseline, relying purely on spatial and morphological tissue features.
H&E + DNA Methylation	0.95 (Peak)	Indicates a profound biological synergy between structural tissue alterations and epigenetic silencing (e.g., MLH1 promoter hypermethylation).
H&E + All Omics Modalities	Decreased Performance	Demonstrates that indiscriminate data piling introduces statistical noise, feature redundancy, and overfitting.

While AI prediction for non-MSI molecular alterations (including BRAF, KRAS, NRAS, TP53, PIK3CA, CIN, CIMP, TMB, and CMS) has demonstrated promising single-center or exploratory signals, significant generalizability barriers remain. Table 3 provides a structured overview of these non-MSI targets, detailing cohort scale, model architectures, validation frameworks, reported performance, and key biological limitations [29].

**Table 3:** Summary of AI-Based Prediction Pipelines for Non-MSI Molecular Biomarkers and Phenotypes in Colorectal Cancer.

Target Biomarker	Representative Study [Ref]	Cohort Size (N)	Algorithm/Architecture	Validation Setting	Performance (AUROC/Metrics)	Major Limitations and Challenges
BRAF V600E	Gustav et al. [27] Bilal et al. [9]	N = 1912 (Multicenter) N = 526 (TCGA)	Multi-target Vision TransformerResNet34 + IDaRS	External Multicenter Internal Cross-Val	AUROC = 0.78 AUROC = 0.79	Heavy morphological overlap and confounding by CIMP-high and MSI-H microenvironments (TILs, mucin).
KRAS	Zhang et al. [32] Jang et al. [33]	N = 435 N = 534	CLAM (Weakly supervised) Inception-v3	Single-center Internal External Transfer	AUROC = 0.896 Performance Decay	Single-center models overfit to local laboratory artifacts; significant performance drop when tested on external cohorts.
NRAS	Zhang et al. [32] Niehues et al. [16]	N = 435 N > 2000	CLAM Self-Supervised MIL	Single-center Internal External Multicenter	AUROC = 0.937 Below Clinical Grade	Low baseline mutation prevalence (<5%) drives high metric variance; fails to generalize externally.
TP53	Bilal et al. [9] Jang et al. [33]	N = 526 (TCGA) N = 534	ResNet34 + HoVer-NetInception-v3	Internal Cross-Val Local Hold-out	AUROC = 0.73 Moderate Predictability	Moderate performance; nuclear pleomorphism overlaps with generalized chromosomal instability.
PIK3CA	Niehues et al. [16] Jang et al. [33]	N > 2000 N = 534	Transformer/SSLInception-v3	External Multicenter Local Hold-out	AUROC < 0.65 Inconsistent	Lacks a distinct, reproducible histopathological footprint on routine H&E.
Chromosomal Instability (CIN)	Hyeon et al. [28] Bilal et al. [9]	N = 526 (TCGA) N = 526	Nuclear Morphometry + CNNWeakly Supervised MIL	Internal Cross-Val Internal Cross-Val	Aneuploidy Score Pred. AUROC = 0.83	Complex genomic state; relies heavily on nuclear pleomorphism and tumor cellularity estimation.
CpG Island Methylator (CIMP)	Bilal et al. [9] Tsai et al. [30]	N = 526 (TCGA) N = 500+	IDaRSMOMA Multi-omics Fusion	Internal Cross-Val External Hold-out	AUROC = 0.79 Moderate	Epigenetic phenotype strongly correlated with BRAF and MSI; difficult to isolate as an independent morphology.
Tumor Mutational Burden (TMB-H)	Shimada et al. [25]	N = 183	CNN + TIL Quantification	Internal Random Sets	AUROC = 0.934	High reliance on TIL density; difficult to distinguish MSS/TMB-H from standard MSI-H cases without sequencing.
Consensus Subtypes (imCMS)	Sirinukunwattana et al. [34]	N = 1200+	Deep Learning imCMS Classifier	Multicenter and Biopsies	AUROC = 0.84–0.85	Intratumoral transcriptional heterogeneity can blur regional classification boundaries.

## 5. Prediction of *BRAF*, *KRAS*, *NRAS* Mutations

Predictive modeling of MAPK pathway alterations from routine H&E-stained WSIs has progressed from conventional CNNs, such as Inception-v3 baselines operating on 360 × 360 pixel patches [33], to self-supervised foundation architectures [32,35]. Modern preprocessing pipelines digitize slides at 20× magnification, apply Vahadane or Macenko color normalization, and execute automated tissue segmentation via Otsu thresholding or specialized ResNet18 and Swin Transformer tissue filters [9,15,32]. To overcome the burden of labor-intensive patch annotation, weakly supervised multiple-instance learning (MIL) frameworks—including IDaRS, which couples an adapted ResNet34 ranking engine with HoVer-Net nuclear lineage mapping—are used to aggregate tile-level probabilities [9]. State-of-the-art pipelines deploy modality-specific, self-supervised vision transformers (ViTs) and pathology-specific encoders (e.g., UNI, CTransPath) decoded via clustering-constrained attention-based MIL (CLAM) or encoder–decoder structures [27,32,35]. To reduce extreme hardware constraints, sequential paradigms such as DPSeq utilize two-dimensional bidirectional long short-term memory (BILSTM2D) networks to compress parameters to a lightweight ResNet50 scale while capturing global spatial context [15]. Concurrently, decentralized swarm learning (SL) deploys blockchain coordination to jointly optimize multicenter algorithms without sharing raw gigapixel data [22].

### 5.1. BRAF Mutation: Predictable but Biologically Entangled with MSI/CIMP

*BRAF* mutation status is among the more predictable non-MSI targets, but its interpretation remains complex. Bilal et al. reported an AUROC of 0.79 for *BRAF* mutation prediction [9]. Guo et al. and Cen et al. reported strong *BRAF* predictive performance using Swin-T and DPSeq, respectively, including in external validation settings [14,15]. Niehues et al. found that BRAF prediction could reach clinical-grade performance with self-supervised, attention-based multiple-instance learning, and that multi-input models improved external generalizability [16]. Furthermore, Ruusuvuori et al., discussing a large, fully transformer-based CRC biomarker study, reported AUROC values of 0.86 for *BRAF* and 0.75 for *KRAS* in a multicentric cohort [35].

However, Gustav et al. provided a key interpretive caution. In a multicenter cohort utilizing a multi-target transformer, MSI, hypermutation, BRAF, and RNF43 were among the best-predicted targets; however, highly predictable alterations were often correlated with MSI and shared MSI-associated morphology [27]. To understand this algorithmic behavior, it is necessary to explore the profound biological entanglement between *BRAF V600E* mutations and the CpG island methylator phenotype (CIMP). In sporadic CRC, BRAF V600E mutations frequently drive the serrated neoplasia pathway, inducing widespread promoter hypermethylation (CIMP-high) [1,2,4]. When this epigenetic silencing affects the MLH1 promoter, the tumor rapidly acquires dMMR and MSI-H status [1,2,4]. Consequently, approximately 30% of sporadic dMMR/MSI-H cases harbor a *BRAF* mutation [4,5,6]. Because the dMMR state dictates the dominant histological presentation—characterized by dense tumor-infiltrating lymphocytes, Crohn-like lymphoid reactions, and mucinous or medullary differentiation—an H&E-based AI model trained to detect *BRAF* is heavily confounded [4,27]. The deep learning architecture may not be identifying a unique, intrinsic *BRAF*-mutant cytological signature, but rather recognizing the generalized morphological field of an immune-rich, CIMP-high/MSI-H microenvironment [27]. This confounding presents significant challenges for clinical translation. If a model relies on MSI-driven immune features to predict *BRAF* mutations, it risks generating false positives in *BRAF* wild-type MSI-H tumors (such as those associated with Lynch syndrome), and false negatives in BRAF-mutant tumors that remain microsatellite stable (MSS) and lack dense lymphocytic infiltrates. In clinical practice, this distinction is critical: AI-based BRAF prediction may enrich cases for confirmatory testing, but it cannot currently replace mutation-specific molecular testing when therapeutic decisions, such as the application of BRAF/EGFR inhibitor combinations, require exact genotyping. Future computational studies must address this entanglement directly by developing co-occurrence-aware frameworks or by training conditional AI models that predict BRAF status independently within MSS and MSI-H patient subgroups.

### 5.2. KRAS and NRAS: Promising Signals with Persistent Generalizability Concerns

*KRAS* and *NRAS* mutations lead to the continuous activation of the MAPK signaling pathway, which drives uncontrolled cellular proliferation and anti-apoptosis independently of upstream EGFR signals. While historically considered difficult to identify without molecular sequencing, these mutations do manifest with subtle but recognizable morphological phenotypes on conventional H&E slides. Histologically, *KRAS*-mutated colorectal cancers frequently present with altered glandular architectures—specifically serrated, papillary, or micropapillary growth patterns—and are often associated with mucinous differentiation and a solid tumor component. These specific structural aberrations and distinct cellular differentiation patterns provide the morphological textures and spatial architectures that deep learning networks exploit to infer mutation status.

*KRAS* prediction remains less mature. Bilal et al. reported an AUROC near 0.60 for *KRAS*, indicating limited discrimination [9]. Jang et al. showed that *APC*, *KRAS*, *PIK3CA*, *SMAD4*, and *TP53* mutations could be predicted from CRC H&E slides to varying degrees, but external generalization was inconsistent; *KRAS* predictive performance dropped when TCGA-trained models were applied to the Seoul St. Mary’s Hospital cohort, improving only when the datasets were combined [33]. Niehues et al. similarly concluded that *KRAS*, *NRAS*, and *PIK3CA* predictions remained below clinical-grade performance in their large multicenter evaluation [16].

In stark contrast to these multicenter limitations, Zhang et al. reported highly optimistic data, achieving an AUROC of 0.89 for *KRAS*, 0.94 for *NRAS*, 0.99 for *BRAF*, and approximately 0.99 for HER2 3+ versus non-3+ classification using CLAM models [32]. However, it is important for readers to recognize the sharp contrast between these metrics and the modest performance observed in the aforementioned multicenter evaluations [16,33]. The results reported by Zhang et al. were derived exclusively from a single-center study utilizing internal cross-validation on a limited cohort (435 CRC patients, with explicitly small *NRAS*-positive and *BRAF*-positive subsets), and they completely lack external validation [32]. From a pathologist’s perspective, this discrepancy illustrates a classic pitfall in computational pathology: a model trained and tested internally within a single institution frequently captures local laboratory artifacts—such as tissue processing techniques, fixation protocols, scanner properties, and population-specific case mixes—rather than the true, generalizable morphological signatures of a mutation [32]. Until these high-performance signals are rigorously replicated across diverse, independent cohorts, *KRAS* and *NRAS* predictions from H&E remain strictly exploratory. For anti-EGFR therapy selection, these targets must remain molecularly confirmed.

The difference between single-center optimism and multicenter reality highlights the diagnostic fragility of H&E-based *MAPK* gene prediction. High AUROCs achieved in restricted single-center studies frequently capture local pre-analytical noise, such as slide preparation protocols, scanner color profiles, and institution-specific tissue handling, rather than true mutation-specific cytological features. Furthermore, low baseline mutation prevalence (particularly for *NRAS* and non-*V600E BRAF* variants) inflates predictive variance in small cohorts. Crucially, algorithms attempting to predict *BRAF* are heavily confounded by the shared CIMP-high/MSI-H phenotype; models rely heavily on dense lymphocytic infiltrates and mucinous pools, leading to poor specificity in MSS *BRAF*-mutated tumors. Until architectures incorporate multi-scanner stain normalization, strict external zero-shot validation, and MSI-stratified subgroup testing, AI predictions for *KRAS*, *NRAS*, and *BRAF* remain strictly investigational tools (Table 4).

## 6. Prediction of Broader Molecular Phenotypes

### 6.1. Chromosomal Instability and Nuclear Morphometry

CIN is the predominant pathway of colorectal carcinogenesis, characterized by widespread aneuploidy, persistent loss or gain of whole chromosomes, and somatic copy number alterations. This pathway is classically driven by the sequential accumulation of mutations in tumor suppressor genes and oncogenes, such as *APC*, *TP53*, and *SMAD4*. On conventional H&E-stained slides, this severe genomic disruption translates directly into profound cytonuclear and architectural chaos. CIN-high tumors typically display marked nuclear pleomorphism, hyperchromatic nuclei, prominent nucleoli, increased and atypical mitotic figures, and extensive architectural distortion, often accompanied by central glandular necrosis. These pronounced, visually striking features of cellular atypia serve as the fundamental histopathological foundation that allows AI models to accurately differentiate CIN-high from genomically stable tumors.

Bilal et al. reported an AUROC of 0.83 for CIN prediction and found that CIN-associated predictive regions were enriched for neoplastic epithelial and mesenchymal cells, exhibiting relatively lower inflammatory and necrotic proportions [9]. Hyeon et al. directly investigated CIN using tile-based and nucleus-based approaches in the TCGA CRC cohort, analyzing more than 350,000 tumor tiles and nearly 2.7 million tumor nuclei. Their study demonstrated that histological features can predict aneuploidy scores and that combining CNN-derived and morphological nuclear features improves nucleus-based classification [28]. Furthermore, nuclear anisokaryosis and intensity variation emerged as interpretable correlates, reinforcing the biological link between chromosomal instability and morphometric nuclear phenotypes [28].

### 6.2. Tumor Mutational Burden Associated with Immune-Rich Morphology

Tumor mutational burden (TMB) represents the total number of somatic substitutions, insertions, and deletions per megabase within the coding region of a tumor genome. High TMB leads to the generation of numerous immunogenic neoantigens, rendering these tumors highly susceptible to immune checkpoint inhibitors. While high TMB strongly overlaps with the MSI-H phenotype, it can also occur in a subset of microsatellite-stable (MSS) tumors. Histologically, the sheer volume of neoantigens elicits a robust, localized immune response. On conventional H&E WSIs, TMB-H tumors are classically characterized by dense populations of tumor-infiltrating lymphocytes (TILs) and a pronounced peritumoral Crohn-like lymphoid reaction. Deep learning models primarily leverage these spatial immune infiltrate patterns—quantifying the density, distribution, and organization of lymphocytes—as morphological proxies to predict high mutational burden.

Although TMB-H is closely related to immunogenicity, it is not identical to MSI. Shimada et al. evaluated CRCs for TMB-H status and found that TILs were significantly associated with high mutational burden. TIL count alone predicted TMB-H with an AUC of 0.910, while a CNN-based H&E model achieved an average AUROC of 0.93 across randomized validation tests [25]. Notably, the model predicted both the MSI-H/TMB-H and MSS/TMB-H subgroups with high accuracy in their internal analyses [25]. This is clinically relevant because MSS/TMB-H CRCs may represent an additional immunotherapy-relevant group beyond conventional MSI screening, although prospective therapeutic validation was not performed in the study.

### 6.3. CIMP and CMS Phenotypes

CIMP and CMS represent integrated molecular phenotypes rather than single-gene alterations. Bilal et al. reported an AUROC of 0.79 for CIMP-high status [9], while Cen et al. and Guo et al. demonstrated that efficient architectures can predict CIMP alongside MSI, *BRAF*, and other biomarkers [14,15]. Tsai et al. developed the MOMA platform to link histopathology images with multi-omics aberrations and prognosis. MOMA predicted MSI with an AUROC of 0.88 ± 0.06 in a TCGA held-out test set and 0.76 ± 0.04 in the NHS-HPFS cohort, predicted BRAF V600E with moderate performance, and identified histological concepts—such as lymphocytes, stroma, mucus, tumor epithelium, and smooth muscle—as key contributors to multi-omics and survival predictions [30]. MOMA also predicted CIMP-high and CMS-related phenotypes with more modest performance, emphasizing that not all molecular states are equally visible in H&E morphology [30].

Nguyen et al. provided a morphology-first anchor for these AI findings. Their weakly supervised segmentation model quantified extracellular mucin-to-tumor area with excellent agreement among pathologists. Mucinous tumors were enriched in CMS1, CMS3, and CMS4, while being essentially absent from CMS2, where average mucin area was 1.8%. MUC5AC expression correlated with *BRAF* mutation status and dMMR, while MUC2 showed distinct clinicopathological associations [26]. Sirinukunwattana et al. extended this approach into image-based CMS classification. Their imCMS classifier achieved an AUROC of 0.84 in TCGA and 0.85 in GRAMPIAN biopsy material, spatially resolved intratumoral heterogeneity, classified RNA-unclassifiable samples, and reproduced expected molecular and prognostic associations [34]. Histological patterns were biologically coherent: imCMS1 exhibited mucin and lymphocytic infiltration; imCMS2 showed cribriform growth and comedo-like necrosis; imCMS3 displayed ectatic, mucin-filled glands with papillary or cribriform components; and imCMS4 demonstrated infiltrative growth, desmoplastic stroma, and tumor budding [34].

### 6.4. TP53 Mutational Analysis

*TP53* prediction occupies an intermediate position in terms of algorithmic performance. Bilal et al. reported an AUROC of 0.73 for TP53 [9], while Jang et al. found TP53 to be one of the better-performing actionable mutation targets in their CRC mutation classifiers [33]. Noguchi et al. provided a related proof-of-principle in ulcerative colitis-associated neoplasia: a CNN trained on H&E patches predicted p53 IHC staining patterns with an accuracy of 0.86–0.91, a sensitivity of 0.73–0.83, a specificity of 0.91–0.92, and an F-measure of 0.77–0.86 [36]. Although this cohort does not represent unselected sporadic CRC, it demonstrates that altered p53 biology can be reflected in glandular and epithelial morphology detectable on H&E.

### 6.5. CLCA1 and Expression-Level Pathomics

Yao et al. extended pathomics toward gene expression inference by predicting *CLCA1* expression in colon adenocarcinoma from H&E features using random forest and XGBoost models. The random forest model achieved an AUROC of 0.85 in the training set and 0.78 in the validation set, and the derived pathomic signatures were linked to prognosis, immune infiltration, mutation patterns, epithelial–mesenchymal transition, and VEGF signaling [37]. Although this specific target is less directly relevant to current CRC molecular decision algorithms than established biomarkers such as MSI, *RAS*, *BRAF*, or HER2, the study broadens the conceptual field: H&E-based AI may infer not only genomic mutations but also complex transcriptomic and microenvironmental states.

## 7. What Is the AI Actually “Seeing”?

### 7.1. Immune Microenvironment

**Figure 3:**
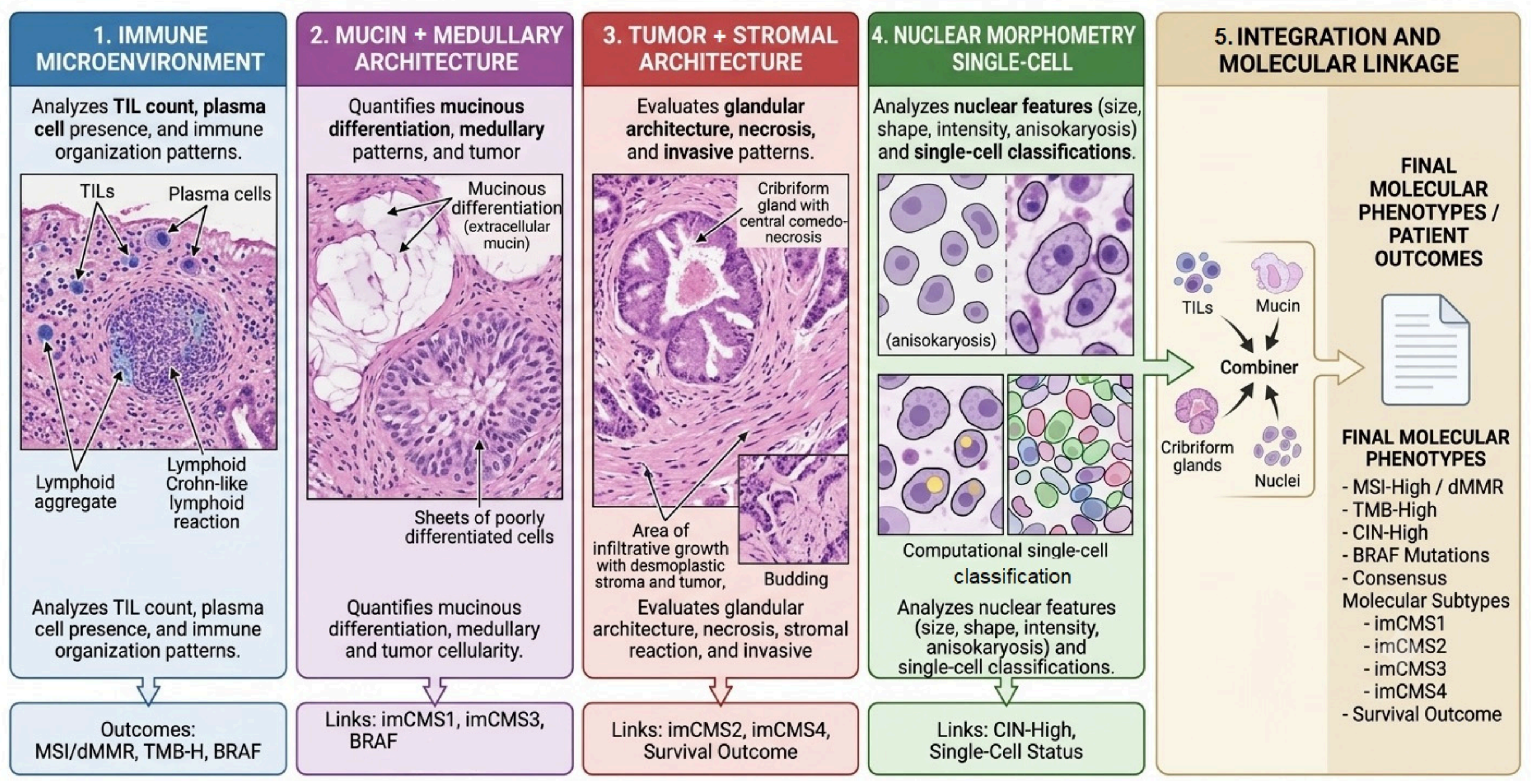
Visual substrates in CRC for molecular phenotyping.

Beyond intrinsic tumor morphology, the most reproducible visual substrate for these predictive models is the immune microenvironment. MSI phenotype, MMR status and TMB prediction models consistently leverage lymphocytes, plasma cells, Crohn-like lymphoid reactions, and immune-rich tumor regions [9,17,18,23,24,25,30,31]. Bilal et al. found high proportions of tumor-infiltrating lymphocytes and necrotic tumor cells in MSI-associated predictive tiles, as well as high lymphocyte abundance in hypermutation-associated regions [9]. The same authors benchmarked a foundation model that identified plasma cell-associated features as key differentiators between MSI and MSS [18]. Shimada et al. showed that TIL count predicted TMB-H with a high AUROC and that their CNN likely learned these immune microenvironmental features [25]. Similarly, Tsai et al. demonstrated that the MOMA platform utilized lymphocytes, stroma, mucosa, and tumor regions for MSI prediction [30]. Fundamentally, because both dMMR and a high mutational burden generate highly neoantigenic tumors, their histological manifestations are not limited to intraepithelial lymphocytes. Instead, they encompass broader spatial immune organization, including lymphoid aggregates, lymphoplasmacytic infiltration, and complex tumor–stromal interactions (Figure 3).

### 7.2. Mucin, Medullary Architecture, and Poor Differentiation

Mucinous differentiation is a recurring but nonspecific signal. MSI-associated CRCs frequently exhibit mucinous or medullary morphology; however, extracellular mucin also appears in CMS3 and CMS4 contexts and may reduce overall tumor cellularity [26,34]. Nguyen et al. demonstrated that the mucin-to-tumor area ratio is a quantifiable phenotype associated with CMS classification and mucin gene expression [26]. Sirinukunwattana et al. showed that mucin and lymphocytic infiltration are characteristic of imCMS1, while ectatic, mucin-filled glands help define imCMS3 [34]. Furthermore, Gustav et al. cautioned that *BRAF* and other targets may be predicted partly because they share this MSI-like, mucinous, and immune-rich morphology [27]. For the pathologist, this is a familiar diagnostic dilemma: mucinous morphology is informative but not determinative. Although AI may quantify mucin more reproducibly than the human eye, its interpretation still requires molecular context (Figure 3).

### 7.3. Tumor and Stromal Architecture, Necrosis and Tumor Budding

Beyond immune infiltrates and mucinous features, AI models exploit glandular architecture, necrosis, stromal reactions, and invasive patterns. For example, imCMS2 was associated with cribriform growth and comedo-like necrosis, whereas imCMS4 was characterized by infiltrative growth, desmoplastic stroma, and tumor budding [34]. Tsai et al. found that tumor-associated stroma and interactions between carcinoma cells and smooth muscle contributed significantly to survival outcomes and multi-omics predictions [30]. Similarly, Bilal et al. showed that distinct molecular phenotypes were associated with varying proportions of neoplastic epithelial, mesenchymal, inflammatory, and necrotic cells [9]. Collectively, these observations suggest that AI is capturing complex spatial tissue organization rather than relying solely on isolated cytological features (Figure 3).

### 7.4. Nuclear Morphometry and Single-Cell Interpretation

Hyeon et al. demonstrated that CIN prediction benefits from nuclear features, including size, shape, intensity, and anisokaryosis [28]. Nowak et al., while utilizing MMR IHC rather than H&E, developed AIMMeR for single-cell MMR status classification and achieved an AUROC of 0.98 against pathologist ground truth in independent CRC trial cohorts [38]. Schoenpflug et al. similarly showed the importance of cell-level quantification of tumor and non-neoplastic tissue for accurate molecular interpretation [20]. Collectively, these studies indicate that future AI systems may combine whole-slide phenotyping with specific cell counts, nuclear morphometry, tumor purity estimation, and spatial biomarker scoring (Figure 3).

## 8. Limitations and Pre-Analytical Challenges

Generalizability remains the central barrier. Domain shift arises from staining protocols, scanner optics, magnification, image compression, fixation, tissue processing, section thickness, artifacts, case mix, population differences, and tumor sampling. Bustos et al. explicitly identified project, patient spot, and TMA glass as bias variables and used adversarial bias rejection to reduce spurious learning [23]. Chang et al. showed that internal performance may not transfer directly to the TCGA cohort, with magnification and cohort differences affecting the results [12]. Jang et al. observed reduced external performance for TCGA-trained mutation classifiers when applied to SMH cases [33]. Furthermore, Zhang et al. explicitly cautioned that their KRAS/NRAS/BRAF/HER2 models were single-center and internally validated only [32].

AI models learn the labels they are given. MSI labels may be derived from IHC, PCR, NGS, MSIsensor, clinical reports, or integrated adjudication. *BRAF* status may be defined by VE1 IHC, PCR, sequencing, or mutation-specific NGS. HER2 status may require IHC scoring and reflex FISH. Nowak et al. showed that lower agreement with MSI PCR could reflect biological discordance between MMR and MSI testing rather than an AI failure [38]. Therefore, biomarker AI studies must clearly define the reference standard, specify discordance handling, and report whether the labels represent molecular truth, protein expression, or clinical diagnostic categories.

Many models are trained exclusively on surgical resections. Biopsies, however, are smaller, more fragmented, and more artifact-prone; they may also lack an invasive front, a desmoplastic interface, or representative mucinous and immune components. Sirinukunwattana et al. demonstrated imCMS classification in rectal biopsy material [34], and Ruusuvuori et al. highlighted transformer-based performance on biopsy MSI prediction, although they also noted that clinical AI solutions for MSI have historically shown poorer performance on biopsies [35]. Deepath-MSI was developed on surgically resected primary CRC specimens and was explicitly framed as a prescreening tool requiring strict quality control [17]. A clinically deployable model must specify the accepted specimen type and must abstain from prediction when tumor content is insufficient.

The prevalence of MSI-H/dMMR is lower than that of MSS/pMMR in many CRC populations. Consequently, even high-performing models may yield a modest positive predictive value (PPV) when deployed as population screening tests. Deepath-MSI achieved a PPV of 34.7% in real-world validation despite a very high negative predictive value (NPV), reflecting this low-prevalence setting [17]. This is not a defect if the intended use is rule-out prescreening. However, it means that AI-positive cases will still require confirmatory molecular testing, and institutions must calibrate their decision thresholds according to local clinical risk tolerance.

Gustav et al. identified a major conceptual limitation: highly predictable molecular targets may appear so simply because they co-occur with MSI and share MSI-associated morphology [27]. This confounding affects *BRAF*, *RNF43*, hypermutation, and other alterations enriched in MSI-like tumors. Without co-occurrence-aware analysis, a model may appear to predict a gene-specific alteration while actually recognizing an immune-rich, mucinous MSI phenotype. Future studies must perform subgroup analyses within the MSI and MSS strata, assess conditional performance, and distinguish direct genotype-specific morphology from correlated molecular ecosystems.

## 9. Clinical Utility and Workflow Integration

While high negative predictive values (NPVs) for MSI phenotype and MMR status prediction have been widely reported in retrospective cohorts, these metrics must be interpreted with clinical caution. Because NPV is inherently prevalence-dependent, a high score in a curated, balanced dataset does not automatically guarantee clinical safety when deployed in real-world populations with varying disease prevalence. At their present stage of development, these deep learning algorithms should be strictly contextualized as potential triage or prescreening tools designed to streamline workflows and flag high-risk cases for rapid assessment. Under no circumstances do these AI predictors currently replace gold-standard, guideline-based confirmatory testing via immunohistochemistry (IHC) or molecular sequencing.

This triage workflow is currently best supported for MSI phenotype and MMR status assessment. Deepath-MSI explicitly proposes rapid, H&E-based prescreening paired with confirmatory testing for predicted MSI-H cases [17]. Foundation model benchmarking for MSI similarly frames AI as a prescreening aid rather than a replacement for standard diagnostics [18]. The AIMMeR pipeline developed by Nowak et al. offers a complementary path by automating the interpretation of MMR IHC at single-cell resolution, thereby reducing pathologist workload while retaining protein-level diagnostic specificity [38]. These strategies are not mutually exclusive; a mature computational molecular pathology workflow could seamlessly integrate H&E prescreening, IHC automation, tumor purity estimation, and reflex NGS testing.

For *BRAF*, *KRAS*, *NRAS*, HER2, *TP53*, and other genetic alterations, the clinical role remains less immediate. AI-based BRAF prediction may enrich cases for confirmatory testing, but it remains highly vulnerable to MSI/CIMP confounding [27]. *KRAS*/*NRAS* prediction remains insufficiently generalizable in multicenter evaluations [16,33], despite promising single-center CLAM results [32]. Similarly, predicting HER2 status requires rigorous validation against established IHC/FISH workflows prior to clinical adoption [32]. Finally, targets such as CIN, TMB, CIMP, CMS, and CLCA1 are scientifically compelling, but they require clearer clinical actionability and prospective validation before real-world implementation [25,26,28,30,34,37].

## 10. Future Perspectives

The transition from retrospective AI studies to clinical molecular pathology requires several critical developments. First, prospective multicenter validation is mandatory. Models must be tested directly within the laboratories where they will be deployed, across variations in local staining protocols, scanner optics, tissue processing, case mix, and reporting workflows. External validation must not be optional; it is the defining threshold between a research classifier and a clinical diagnostic device.

Second, AI tools must report uncertainty and sample adequacy. A case with low tumor cellularity, extensive mucin, prominent necrosis, out-of-focus artifacts, or severe biopsy fragmentation should not receive a misleading binary output. Algorithms must incorporate explicit abstain mechanisms when tissue quality or confidence thresholds are unmet.

Third, interpretability should mature from illustrative heatmaps to quantitative pathology. The most compelling studies link AI predictions to quantifiable biological features, such as immune cells, plasma cells, mucin area, tumor heterogeneity, nuclear morphometry, tumor purity, and CMS-like tissue organization [18,20,24,26,28,30,34]. These explainable features should be evaluated by blinded pathologists and quantified against biologically meaningful compartments.

Fourth, multimodal AI should be selective rather than indiscriminate. Qiu et al. showed that combining H&E with DNA methylation outperformed H&E alone, but that adding all omics modalities indiscriminately reduced performance relative to selected feature fusion [29]. Similarly, Wei et al. showed that integrating clinical features can improve MSI prediction [31]. The future is likely not “all data into one model,” but rather the biologically constrained integration of H&E morphology, IHC metrics, clinical variables, tumor sidedness, tumor purity, molecular priors, and selected omics features.

Fifth, decentralized and privacy-preserving training may become central. Swarm learning provides a framework for multicenter collaboration without centralizing sensitive pathology data [22]. This approach is especially relevant for rare alterations such as *NRAS* mutations, *BRAF* variants in specific subgroups, HER2 amplification, POLE-associated TMB-H, and uncommon molecular phenotypes.

Finally, regulatory and laboratory governance must be explicit. Clinical AI requires locked model versions, audit trails, continuous performance monitoring, stain/scanner change management protocols, bias assessment, cybersecurity, failure mode analysis, and seamless integration with the laboratory information system. Ultimately, the model output must be interpretable to the pathologist and actionable to the oncologist, while preserving the primacy of validated molecular tests whenever treatment depends on exact genotype.

## 11. Conclusions

AI-based prediction of molecular alterations from routine H&E-stained CRC slides has advanced from proof-of-concept to credible translational potential, especially for MSI phenotype and MMR status prescreening. The most mature application of AI in colorectal cancer pathology lies in the evaluation of mismatch repair status and microsatellite instability. However, the future clinical deployment of these tools relies on precision in their biological definitions. AI has proven highly adept at recognizing the morphological hallmarks of genomic hypermutation (the MSI-H phenotype) and the localized structural loss of repair proteins (the dMMR state). Yet, as we move toward clinical integration, developers and pathologists must avoid conflating these distinct targets. Future regulatory approvals and clinical workflows will require algorithms to explicitly state whether they are validated to predict protein-level status (pMMR vs. dMMR) or genomic-level instability (MSI-H vs. MSI-L vs. MSS), as this distinction directly dictates the appropriate confirmatory reflex testing. BRAF prediction is promising but frequently reflects MSI/CIMP-associated morphology and must be interpreted in the context of molecular co-occurrence. *KRAS*, *NRAS*, *PIK3CA*, and several other single-gene alterations remain below clinical readiness in broadly validated settings, despite encouraging single-center results. Targets such as CIN, TMB-H, CMS, CIMP, p53-related patterns, tumor purity, *CLCA1* expression, and multi-omics phenotypes demonstrate that routine histology contains a dense morphomolecular signal. The pathologist’s role remains central: AI should augment tissue assessment, prioritize confirmatory testing, identify morphologically and molecularly enriched cases, and accelerate clinical workflows, but it should not replace IHC, PCR, or NGS where validated molecular evidence is required. The future of this field lies in robust, interpretable, regulated, and pathologist-supervised systems that convert routine H&E slides into clinically useful molecular prescreening instruments.

## Figures and Tables

**Table 1:** Reported performance metrics for representative AI-based MSI phenotype and/or MMR status prediction pipelines.

Model/Pipeline [Ref]	Target Biomarker	Tissue Processing and Dataset	Ground-Truth Reference Standard	Cohort/Evaluation Setting	Validation Strategy	Reported Performance Scores and Metrics
EPLA [10]	MSI	Training: Flash-frozen sections (TCGA) Testing: FFPE sections	PCR/Clinical Annotations	Flash-frozen TCGA cohorts External FFPE tissues (pre-fine-tuning)	Internal Cross-Val External Fine-Tuning	AUROC = 0.88 (Frozen internal) AUROC = 0.85 (FFPE External)
IDaRS [9]	MSI	FFPE (Unseen challenge datasets)	IHC/PCR	Unseen challenge datasets (External)	External Zero-Shot	AUROC = 0.98
DPSeq [15]	MSI Hypermutation	FFPE (International validation sets)	PCR/NGS	International validation sets	External Validation	AUROC = 0.92 (AUPRC = 0.68) AUROC = 0.88
Multi-target transformer encoders [27]	MSI Hypermutation BRAF mutations	FFPE (External validation, 1912 patients)	NGS/IHC	External validation (1912 patients)	External Multicenter	AUROC = 0.93 AUROC = 0.88 AUROC = 0.78
xDEEP-MSI [23]	MSI	FFPE (Tissue microarrays, 1788 patients)	IHC/PCR	Tissue microarrays (1788 patients)	Patient-Level Cross-Val	Patient-level AUROC = 0.90
PPsNet [11]	MSI	FFPE (Asian CRC dataset)	PCR	Asian CRC dataset	Hold-Out Validation	Validation Accuracy = 87.3% AUROC = 0.94
WiseMSI [12]	MSI	FFPE (Multicenter Chinese cohort + TCGA)	IHC/PCR	Multicenter Chinese cohort	External Multicenter Cross-Domain Testing	Specificity = 94.7% Sensitivity = 84.7% AUROC = 0.95
PAIP 2020 Top-Ranked System [13]	MSI	FFPE (Challenge benchmark dataset)	IHC	Challenge benchmark	Pre-curated Challenge Set	F1 score = 0.92
Swin-T [14]	MSI	FFPE (TCGA + Macao Cohort)	PCR/IHC	TCGA cross-validation Cross-study validation (MCO to TCGA)	Internal Cross-Val Cross-Study External	AUROC = 0.91 AUROC = 0.90
Deepath-MSI [17]	MSI/dMMR	FFPE (Multicenter test set, 5070 WSIs + Real-world cohort)	IHC and PCR integrated	Multicenter test set (5070 WSIs, 7 cohorts) Multicenter test set (At fixed 95% sensitivity) Real-world validation cohort	External Zero-Shot Real-World Prospective-Like	AUROC = 0.98 Specificity = 91.7% PPV = 69.0% NPV = 99.0% Accuracy = 92.2% Sensitivity = 94.6% Specificity = 90.7% PPV = 34.7% NPV = 99.7%

Note on Metric Comparability: Direct quantitative comparison of AUROC, sensitivity, and accuracy scores across rows is restricted by fundamental differences in ground-truth definitions (IHC vs. PCR vs. NGS), cohort composition (pre-curated challenge tiles vs. unselected clinical WSIs), disease prevalence, and validation strategies (internal cross-validation vs. zero-shot external generalization). Performance metrics should be evaluated within the context of each study’s specific validation framework and intended clinical application. Abbreviations: AUROC, Area Under the Receiver Operating Characteristic curve; AUPRC, Area Under the Precision–Recall Curve; FFPE, Formalin-Fixed Paraffin-Embedded; MCO, Macao Cohort; NPV, Negative Predictive Value; PPV, Positive Predictive Value; TCGA, The Cancer Genome Atlas; WSI, Whole-Slide Image.

**Table 4:** Comparative Limitations of AI Models Predicting MAPK Pathway Alterations.

Limitation Parameter	Single-Center Models (Zhang et al. [32])	Multicenter/Large Cohort Models (e.g., Niehues [16], Gustav [27], Jang [33])
Cohort Size and Prevalence	Small cohorts (N < 500); low absolute count of positive cases (e.g., low *NRAS*/*BRAF* prevalence) causes high variance and overfitting risk.	Large, pooled datasets (N > 1000 to 10,000+); reflects true population prevalence, minimizing metric skew.
Validation Strategy	Internal cross-validation only; fails to test algorithm independence against unseen data sources.	External zero-shot or cross-site validation; rigorously evaluates out-of-distribution performance.
Domain and Stain Shift	Blind to pre-analytical noise; algorithms overfit to institution-specific scanners, stain intensities, and fixation protocols.	Exposed to multi-scanner and multi-laboratory stain variability, revealing true performance degradation.
Biological Confounding	Confounders unadjusted; models mistake shared mucinous/TIL signatures of MSI-H/CIMP-high for direct *BRAF* features.	Stratified analyses reveal *BRAF* prediction drops significantly when tested exclusively within MSS cohorts, uncovering morphological co-dependence.

## Data Availability

No new data were created or analyzed in this study. Data sharing is not applicable to this article.

## Abbreviations

The following abbreviations are used in this manuscript:

AI	Artificial intelligence
AUC	Area under the curve
AUROC	Area under the receiver operating characteristic curve
CIMP	CpG island methylator phenotype
CIN	Chromosomal instability
CLAM	Clustering-constrained attention multiple-instance learning
CMS	Consensus molecular subtype
CRC	Colorectal cancer
dMMR	Deficient mismatch repair
FFPE	Formalin-fixed paraffin-embedded
H&E	Hematoxylin and eosin
IHC	Immunohistochemistry
MIL	Multiple-instance learning
MMR	Mismatch repair
MSI	Microsatellite instability
MSS	Microsatellite stable
NGS	Next-generation sequencing
NPV	Negative predictive value
PCR	Polymerase chain reaction
PPV	Positive predictive value
TIL	Tumor-infiltrating lymphocyte
TMB	Tumor mutational burden
WSI	Whole-slide image
